# Changes in Antibiotic Use and Disruptions to Antimicrobial Resistance Detection in South Africa and Uganda, 2019 – 2020

**DOI:** 10.1017/ash.2024.298

**Published:** 2024-09-16

**Authors:** Amara Fazal, Elizabeth Bancroft, Kieran Hartsough, Cherie Cawood, Patrick Kazooba, George Upenytho, Edward Bitarakwate, Rhoderick Machekano, Carolyn Herzig, Godfrey Woelk

**Affiliations:** Centers for Disease Control and Prevention; National Center for Emerging and Zoonotic Infectious Diseases, US Centers for Disease Control and Prevention, Atlanta, USA; Division of Healthcare Quality Promotion, Centers for Disease Control and Prevention; Elizabeth Glaser Pediatric AIDS Foundation

## Abstract

**Background:** The COVID-19 pandemic disrupted routine health services worldwide, including systems to detect antimicrobial resistance (AR). AR is a mounting global health threat with some studies showing the highest mortality rate from AR infection is in Sub-Saharan Africa (SSA). Antibiotic use is a major contributor to AR. We sought to characterize COVID-19-related changes to antibiotic use and AR detection capacity in two countries in SSA from 2019 to 2020. **Methods:** Health facilities (HF) in South Africa and Uganda were surveyed as part of a larger study assessing disruptions to essential health services in SSA in the context of COVID-19. Modified stratified random sampling of HF by facility level was conducted in regions with high COVID-19 cumulative prevalence. Hospital pharmacists were surveyed to identify perceived changes in antibiotic use. Among facilities with the capacity to detect AR, surveys were conducted with AR laboratory managers to identify perceived changes in staff, equipment, training, and supplies. Descriptive data analysis was conducted using frequencies and proportions. **Results:** A total of 39 HFs in South Africa and 45 HFs in Uganda responded to the antibiotic use survey. Increases in total antibiotic use from 2019 to 2020 were reported by 82% (23/28) of HF in South Africa and 68% (27/40) in Uganda. Increased use of antibiotics for multi-drug resistant bacteria (per World Health Organization Reserve classification) was reported by 36% (9/25) and 38% (8/21) of HFs in South Africa and Uganda, respectively. 19 HFs in South Africa and 12 HFs in Uganda responded to the AR detection capacity survey. HFs in both countries reported decreases in laboratory staff responsible for AR (33% [13/40] in South Africa and 31% [11/35] in Uganda). Decreased availability of reagents and consumables for bacteriology and antimicrobial susceptibility testing was reported by 50% (8/16) and 33% (4/12) of HFs, and decreased availability of specimen collection supplies for bacterial cultures was reported by 41% (7/17) and 42% (5/12) of HFs in South Africa and Uganda, respectively. Diversion of laboratory supplies was reported in both countries (32% [6/19] in South Africa and 25% [3/12] of HF in Uganda). **Conclusions:** HFs in South Africa and Uganda reported increases in antibiotic prescribing, a risk factor for increased AR, concurrently with disruptions in AR detection capacity during the early phases of the COVID-19 pandemic. These findings emphasize the importance of investing in bacteriology and AR testing in SSA and maintaining support during infectious disease pandemics.

